# Baseline CD8 T-cell activation is not associated with survival in ATLL

**DOI:** 10.1186/1742-4690-12-S1-P70

**Published:** 2015-08-28

**Authors:** Graham P Taylor, Lucy Cook, Jana Haddow

**Affiliations:** 1 The National Centre for Human Retrovirology (NCHR), Imperial College Healthcare NHS Trust, St. Mary's Hospital, London, UK

## 

Adult T-cell lymphoma/leukaemia (ATLL) is known to be associated with poor immune response. Currently, it is not clear whether this is contributory to, or the consequence of the development of ATLL. In the National Centre for Human Retrovirology (NCHR) in London, we measured CD8/DR expression (a marker of T-cell activation) at ATLL presentation in our cohort of patients and compared these findings with a control population of aged-matched patients with myelopathy (HAM) and asymptomatic carriers (AC). The normal range for CD3+ CD8+ HLA-DR in HTLV-1 uninfected adults is 5.7% – 38.2%. The median overall survival status in our cohort of patients with ATLL (n = 53) is 9 months for acute subtype (range 3-22 months), lymphoma subtype 13.9 months (range 1-135 months), chronic subtype 64 months (range 8-144 months) and smouldering subtype 115 months. The median CD3+ CD8+ HLA-DR for AC was 25% (range 11-76%, n=20), 44% HAM (range 19-80%, n=20) and 45% for all ATLL subtypes (range 9-87%, n=26). By ATLL subtype: acute 58% (range 22-72%, n=5), lymphoma 43.5% (range 11-80%, n=10), chronic subtype 41% (range 23-60%, n=9), cutaneous 50% (n=1), smouldering subtype 46% (n=1). These data show that there is a significant increase in CD3+CD8+ HLA-DR expression in all ATLL subtype compared with AC and similar to that observed in patients with HAM. CD3+CD8+ HLA-DR expression is most marked in the acute subtype which is also associated with shortest survival. Despite evidence of CD8 T-cell activation this does not appear to be protective or associated with improved survival.

